# Effects of Vine Water Status on Malate Metabolism and γ-Aminobutyric Acid (GABA) Pathway-Related Amino Acids in Marselan (*Vitis vinifera* L.) Grape Berries

**DOI:** 10.3390/foods12234191

**Published:** 2023-11-21

**Authors:** Zhennan Zhan, Yanxia Zhang, Kangqi Geng, Xiaobin Xue, Alain Deloire, Dongmei Li, Zhenping Wang

**Affiliations:** 1 School of Life Sciences, Ningxia University, Yinchuan 750021, China; znzhan@126.com (Z.Z.); zhangyanxiaedu@163.com (Y.Z.);; 2 Ningxia Wine and Desertifcation Control Vocational and Technical College, Yinchuan 750199, China; 3 Shanxi Academy Agricultural Sciences, Pomology Institute, Shanxi Agricultural University, Taiyuan 030006, China; 4 School of Agriculture, Ningxia University, Yinchuan 750021, China; 5 Department of Biology-Ecology, L’Institut Agro, University of Montpellier, 34060 Montpellier, France; alain.deloire@supagro.fr

**Keywords:** grape, water stress, malate, amino acid, fruit quality

## Abstract

Malic acid is the predominant organic acid in grape berries, and its content is affected by abiotic factors such as temperature (fruit zone microclimate) and water (vine water status). The objectives of this study were to explore the potential mechanisms behind the effects of vine water status on the biosynthesis and degradation of berry malic acid and the potential downstream effects on berry metabolism. This study was conducted over two growing seasons in 2021 and 2022, comprising three watering regimes: no water stress (CK), light water stress (LWS), and moderate water stress (MWS). Compared to CK, a significantly higher level of malic acid was found in berries from the MWS treatment when the berry was still hard and green (E-L 33) in both years. However, water stress reduced the malic acid content at the ripe berry harvest (E-L 38) stage. The activities of NAD-malate dehydrogenase (NAD-MDH) and pyruvate kinase (PK) were enhanced by water stress. Except for the E-L 33 stage, the activity of phosphoenolpyruvate carboxylase (PEPC) was reduced by water stress. The highest phosphoenolpyruvate carboxykinase (PEPCK) activity was observed at the berry veraison (E-L 35) stage and coincided with the onset of a decrease in the malate content. Meanwhile, the expression of *VvPEPCK* was consistent with its enzyme activity. This study showed that water stress changed the content of some free amino acids (GABA, proline, leucine, aspartate, and glutamate), two of which (glutamate and GABA) are primary metabolites of the GABA pathway.

## 1. Introduction

Sugars, organic acids, and aromatic precursors are important compounds of berry composition that influence fruit flavors and wine aromatic profiles [1]. The metabolism of these compounds in the berries of cultivated grapevines depend on many factors, such as the site (climate and soil), grape variety, viticultural practices, and the level of maturation [2,3]. Among the abiotic factors affecting grapevine and berry physiology, water is one of the main environmental factors that interact with temperature and light at the fruit zone level (microclimatic) [4,5]. Moreover, grapevines are widely planted in semi-arid and arid regions with high evaporation rates and a general shortage of water. Many studies have demonstrated that moderate water stress can effectively improve grape quality while saving irrigation water [4,6,7,8].

In grapes, organic acids are responsible for the titratable acidity, which is one parameter used to evaluate fruit quality [9]. Tartaric and malic acids are the primary acids found in berries, comprising over 60% of the total acidity [10,11]. They also contribute to the pH of the must and wine during vinification and subsequent aging of the wines. Unlike tartaric acid, malic acid is ubiquitously distributed in the plant kingdom and fulfills many distinct and important physiological roles such as glycolysis, the tricarboxylic acid (TCA) cycle, gluconeogenesis, the glyoxylate cycle, and the synthesis of complex secondary metabolites [12]. In general, the accumulation of malic acid in fruits is determined by intricate interactions involving acid synthesis, degradation, and vacuolar storage [13]. The malic acid in grapes accumulates before veraison, and its level decreases continuously during the subsequent ripening process. Previous studies have shown that phosphoenolpyruvate carboxylase (PEPC), NAD-malate dehydrogenase (NAD-MDH), phosphoenolpyruvate carboxykinase (PEPCK), NADP-malic enzyme (NADP-ME), and pyruvate kinase (PK) are intimately involved in the synthesis and degradation of malic acid in fruit [14]. Vine water status has been proven to influence the formation of grape berry acidity in fruit growth and development [8]. Moreover, in many cases, a high temperature was accompanied by lower titratable acidity (TA) of the grape berry at the harvest, which was attributed to the net loss of malate [15,16]. However, little is known about the regulation of malate metabolism and downstream metabolic pathways under water stress, and its molecular basis is still unclear.

Amino acids not only act as osmoprotectants and regulatory substances for plant growth and development but are also important for responding to abiotic stresses [17]. Specifically, arginine, glutamic acid, proline, and GABA are known as abiotic stress responses. γ-aminobutyric acid (GABA) is a non-protein amino acid involved in primary and secondary metabolite synthesis since it is an integral intermediate in nitrogen metabolism and amino acid biosynthesis. The GABA pathway is considered to bypass the tricarboxylic acid cycle (TCA cycle) during photosynthesis and respiration [18], which is closely related to amino acid metabolism. In addition, studies have shown that GABA levels can rapidly increase in response to a range of abiotic stresses [19]. However, few reports exist on the effects of vine water status on the amino acid contents in the GABA pathway in grapes.

Ningxia, one of China’s major wine-producing regions, is facing challenges related to high evaporation rates and a general shortage of water. Despite numerous studies on the effects of water on vine physiology, berry development, and composition, more research is needed to understand the impact of vine water status on the key enzymes involved in berry malic acid metabolism. Therefore, the aim of this study was to investigate how vine water status and water stress affect the organic acid content of grape berries and to explore the changes in gene transcription levels and key enzymes involved in malic acid metabolism. These findings will contribute to a better understanding of the regulation of malic acid and its potential downstream effects on berry metabolism under water stress, with the hope of providing a basis for future studies, including grapevine genetic selection.

## 2. Materials and Methods

### 2.1. Climatic Conditions and Experimental Design

**Figure 1:**
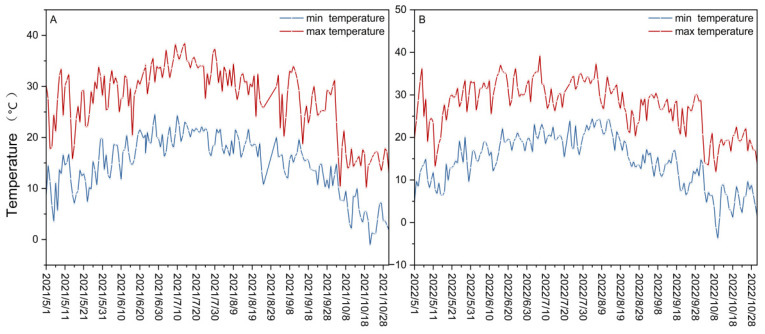
Variations in the minimum and maximum temperature (°C) in the years 2021 (**A**) and 2022 (**B**), beginning in May and ending in October of each year.

The experiment was conducted in 2021 and 2022 at the water physiology and water-saving cultivation post-test base of the National Grape Industry Technology System (CARS-29-zp-3) located in the Yuquanying test field in Yinchuan, Ningxia, China (38°14′25″ N, 106°01′43″ E). The region is situated in the warm temperate zone, characterized by a continental arid and semi-arid monsoon climate, with an average annual temperature of 8.7 °C and abundant solar radiation. From May to October 2021 and May to October 2022, the daily maximum temperature ranged from 10.1 to 38.5 °C and 11.8 to 39.3 °C, respectively. The daily minimum temperature in 2021 ranged from −1.1 to 24.6 °C, and in 2022, it ranged from −3.8 to 24.5 °C (Figure 1A,B). The total rainfall during the test period was 109.045 mm and 154.850 mm in 2021 and 2022, respectively. The monthly rainfall profiles during the June–September period of 2021 and 2022 differed significantly, with the highest monthly precipitation occurring in September and July, respectively (Supplement Table S1). The total rainfall and temperature were measured using automatic weather stations.

A field experiment was conducted on six (seven)-year old own-rooted ‘Marselan’ (*Vitis vinifera* L.cv) wine grapes. All the grapevines were pruned into a cup-shape with five fruit-bearing branches. All the agronomic practices, except for the irrigation volume, were kept uniform. The grapevines were planted in east–west rows with a row spacing of 3 m and a plant spacing of 0.6 m. The soil was a light gray calcareous soil and aeolian sandy soil. The experiment used a randomized block design with three treatments: no water stress (CK), light water stress (LWS), and moderate water stress (MWS), with three replicates.

Water was delivered through a drip irrigation system with a flow rate of 0.6 L·h^−1^, which was controlled by a valve at both ends of each drip line. The irrigation time was adjusted timely based on rainfall and water evaporation to effectively control the irrigation amount (Supplement Table S1). Water stress was applied to the grapevines on 18 June 2021 and 26 June 2022 (E-L 29). The pre-dawn leaf water potential (Ψ_PD_) in the no water stress was controlled at 0 MPa ≥ Ψ_PD_ ≥ −0.2 MPa. The light water stress was controlled at −0.2 MPa ≥ Ψ_PD_ ≥ −0.4 MPa, and the moderate water stress was controlled at −0.4 MPa ≥ Ψ_PD_ ≥ −0.6 MPa according to the thresholds established by Wang et al. [20]. The berries were collected at four stages of the Eichhorn–Lorenz (E-L) scale established by Coombe [21]. E-L 33, 35, 37 (berry not quite ripe), and 38. 200 berries were randomly selected from each treatment at different positions of the bunch (top, middle, bottom, inner, and outer sides). A total of 100 berries were used to determine the physical–chemical indicators, and the remaining berries were frozen in liquid nitrogen and stored at −80 °C for RNA extraction and enzyme activity determination.

### 2.2. Water Status and Phytochemicals of the Grape Berries

Ψ_PD_ was measured every 10 days using a 3005 plant water potential pressure chamber from Soil Moisture Equipment, USA. Five to six leaves (nodes six to eight from the shoot tip) without disease or physical injury were picked from each treatment and measured according to Deloire et al. [22]. At the E-L 38 stage, twenty berries were squeezed to extract juice, which was used to determine the total soluble solids (TSS, as degrees °Brix) using a WYT-32 hand-held sugar refractometer from Quanzhou Zhongyou Optical Instrument Co Ltd., Fujian, China. The titratable acidity (TA) (g·L^−1^ expressed as tartaric acid equivalent) was measured by titrating the fresh juice with 0.05 N of NaOH up to a pH of 8.1. The total anthocyanins content was evaluated using the pH-differential method developed by Lian et al. [23] and measured using the method of Xu et al. [24].

### 2.3. Organic Acids, Enzyme Extraction, and Activity Assay

For the extraction and assay of organic acids and enzyme activity, berries without the rachis and seeds were ground into a fine powder using liquid nitrogen. Wu et al.’s methods with minor modifications were applied to extract and measure the organic acids [25]. The organic acids were extracted from 0.2 g of powder in a final volume of 2.0 mL of distilled water, and the mixture was kept in an ultrasonic bath at 20 °C for 30 min. After 10 min at 10,000× *g*, the supernatant was filtered through a 0.45 µm filter membrane. The content of organic acids (tartaric, malic, citric, fumaric, and succinic acid) was investigated using a thermo UltiMate 3000 high-performance liquid chromatography (HPLC) system (Thermo Fisher Scientific, Shanghai, China) with a C18 column (Hypersil GOLD, 4.6 mm × 250 mm, 5 µm) and a UV detector at 210 nm. The flow rate was 0.8 mL·min^−1^, with a mobile phase consisting of 97% 10 mM KH_2_PO_4_ buffer with a pH of 2.7 and 3% methanol with an injection volume of 10 µL. The column temperature was maintained at 35 °C for 34 min. Organic acid standards used for high-performance liquid chromatography (HPLC) analysis were purchased from Yuan Ye Bio−Technology Co., Ltd (Shanghai, China).

Extraction of PEPC, PEPCK, and PK was performed using the method described by Sweetman et al. [26] with slight modifications. A total of 2 g powder was ground in an ice bath with 20 mL of pre-cooled extraction buffer containing 500 mM pH 8.5 Tris-Cl, 200 mM KCl, 20 mM MgCl_2_, 10 mM EDTA, 8% (*w*/*v*) PEG-4000, 8 mM cysteine-HCl, 5 mM dithiothreitol (DTT), 2% (*w*/*v*) polyvinylpolypyrrolidone (PVPP), 0.25% (*w*/*v*) bovine serum albumin (BSA), 0.5 mM phenylmethylsulfonyl fluoride (PMSF), and 0.5 mM p-aminobenzamidine. The resulting homogenate was transferred to a 50 mL centrifuge tube and centrifuged for 5 min at 3000× *g*. The supernatant was then transferred to a new centrifuge tube, and PEG-4000 was added to a final concentration of 55% (*w*/*v*). The precipitated proteins were resuspended in 2 mL of resuspension solution (5 mM pH 7.0 Tris-Cl, 20 mM MgCl_2_, 10 mM EDTA, 5 mM DTT, 3% (*w*/*v*) Triton X-100, 0.5 mM PMSF, and 0.5 mM ρ-aminobenzamidine) and centrifuged again at 5000 rpm for 5 min. The resulting supernatant was used immediately as a crude enzyme for enzyme activity assays. The enzyme activities of PEPC, PEPCK, and PK were determined following the method described by Sweetman et al. [26].

Extraction of NADP-ME and NAD-MDH was performed according to Hirai and Ueno [27], with slight modifications. Grape pulp (5 g) was weighed and ground in a pre-cooled mortar with 15 mL of extraction solution containing 50 mM pH 7.6 Hepes–Tris, 250 mM sorbitol, 10 mM MgSO_4_, 125 mM KCl, 5 mM EDTA, 2 mM PMSF, 2% polyvinylpyrrolidone (PVP), 0.2% BSA, and 2 mM DTT. The resulting homogenate was filtered through gauze into a 50 mL centrifuge tube and centrifuged at 1000× *g* for 10 min at 4 °C. The resulting supernatant was collected and centrifuged again at 50,000× *g* for 1 h to obtain the crude enzyme solution. The enzyme activities of NAD-MDH and NADP-ME were determined following the method described by Han et al. [28]. Before starting the reaction, the reaction solution was placed in a water bath at 37 °C for 20 min. The absorbance was read at 340 nm every 10 s for 1 min using a microplate reader (Biotek, Winooski, VT, USA, Synergy LX multi-mode reader) immediately after adding each reaction substrate. The enzyme activity was expressed as nmol·min^−1^·g^−1^ FW (fresh weight), and the detection volume was 200 µL. The reagents for enzyme extraction and activity assay were purchased from Beijing Solarbio Science & Technology Co., Ltd. (Solebao, Beijing, China).

### 2.4. Analysis of Soluble Sugars and Amino Acids Using HPLC

In the present study, there were significant differences in the malic acid content of the berries in 2022 across the four stages; therefore, the sampled berries from the 2022 season were used for the determination of soluble sugars (glucose and fructose) and free amino acids. The soluble sugar extraction and determination methods refer to a previous study [29]. The derivatization of amino acids involved the method of Wang et al. [30]. The amino acid content was investigated using a thermo UltiMate 3000 high-performance liquid chromatography (HPLC) system (Thermo Fisher Scientific, Shanghai, China) and was calculated using amino acid mixed standards and a GABA standard subjected to the same derivatization procedure. The amino acids quantified in the grape berries included aspartate (Asp), glutamate (Glu), glycine (Gly), arginine (Arg), γ-aminobutyric acid (GABA), leucine (Leu), and proline (Pro). The conditions for separating the amino acids were as follows: detector, UV detector (280 nm); column, Hypersil GOLD C18 (250 mm × 4.6 mm, 5 µm); phase A, 10 mM Na_2_HPO_4_/Na_2_B_4_O_7_, pH 8.2; phase B, acetonitrile/methanol/water = 45/45/10 (*v*/*v*/*v*); flow rate, 1.0 mL/min; injection volume, 5 µL; column temperature, 16 °C. The elution program was as follows: 2% B kept for 0.5 min, 2% B to 57% B in 24.5 min, 57% B to 100% B in 0.1 min and kept for 3.4 min, and 100% B to 2% B in 0.1 min then kept for 1.4 min, followed by washing and reconditioning the column. HPLC-grade methanol and acetonitrile, were obtained from Macklin (Shanghai, China). Supplementary Figure S1 shows the chromatograms of the free amino acid analysis.

### 2.5. qRT-PCR Analysis

The expression levels of the key enzyme genes *VvNAD-MDH1*, *VvNAD-MDH2*, *VvNADP-ME1*, *VvNADP-ME2*, *VvNADP-ME3*, *VvPEPC1*, *VvPEPC2*, *VvPEPC3*, *VvPEPCK*, *VvPK1*, and *VvPK2* were measured using real-time fluorescence quantitative PCR (qRT-PCR). To prepare the samples, the rachis and seeds of the grape berries were removed, and the grape pericarp was ground into a powder in liquid nitrogen. The total RNA was extracted using a plant polysaccharide polyphenol RNA kit (DP441, TIANGEN Biotech Co., Ltd, Beijing, China). Then, 1 µg of RNA was reverse-transcribed into cDNA using the Takara PrimeScript TM RT reagent Kit with a gDNA Eraser (Perfect Real Time) Kit (Code No. RR047A, TaKaRa, Dalian, China).

The qRT-PCR was carried out using Takara’s TB Green^®^ Premix Ex Taq TM II (Tli RNaseH Plus) kit (Code No. RR820A, TaKaRa, Dalian, China). Amplification and quantification were performed using the BioRad CFX96 system (BioRad, Hercules, CA, USA). The qRT-PCR was performed using a 20 µL reaction volume, including 10 µL of TB Green Premix Ex Taq II, 0.8 µL of forward primer, 0.8 µL of reverse primer, 1.6 µL of cDNA, and 6.8 µL of RNase-free water. The qRT-PCR conditions were as follows: 95 °C for 30 s, followed by 40 cycles of 95 °C for 5 s and 60 °C for 30 s. *VvGADPH* was used as the internal control. The relative expression levels were calculated using the the 2^−ΔΔCT^ method [31]. The primer sequences used are shown in Supplementary Table S2.

### 2.6. Statistical Analysis

The data were processed and graphed using Microsoft Excel 2010, SPSS 22.0 (IBM, Armonk, NY, USA), and Origin 2023 (Origin Lab, Northampton, MA, USA) software. The PCA (principal component analysis) was performed using Origin 2023. Significance analyses were performed using Tukey’s multiple-comparison test at the 0.05 significance level. All the data were expressed as mean ± standard deviation (SD) and analyzed in triplicate.

## 3. Results

### 3.1. The Ψ_PD_ of ‘Marselan’ under Water Stress

**Figure 2:**
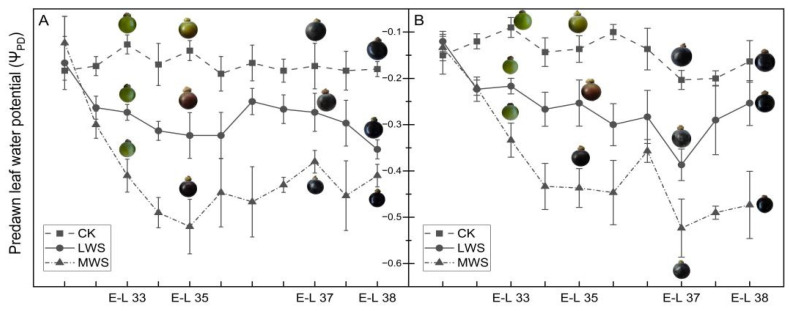
Pre-dawn leaf water potential and berry phenotypic change in 2021 (**A**) and 2022 (**B**) under different vine water statuses.

The Ψ_PD_ of the ‘Marselan’ plant was measured to evaluate its response to varying levels of vine water status. The Ψ_PD_ was significantly lower in the water stress treatments, indicating that the soil water availability in the water stress treatments decreased. The Ψ_PD_ of the ‘Marselan’ plant was controlled within the preset range in 2021 and 2022 by adjusting the irrigation amount during the experiment (Figure 2). The Ψ_PD_ of the leaves before the water stress in both years was approximately the same, ranging from −0.1 to −0.2 MPa. In August 2022, the Ψ_PD_ of the MWS treatment increased slightly due to rainfall. However, timely water control measures were implemented, allowing the Ψ_PD_ to return to the appropriate range for the targeted vine water stress.

### 3.2. Phenotype and Phytochemicals of the Grape Berries under Water Stress

The berry phenotype, 50-berry weight, total soluble solids, titratable acidity, and total anthocyanins content in the developing grape berries are shown in Figure 2 and Table 1. In 2022, the 50-berry weight of the CK treatment was 18.5% and 29.4% higher than that of the LWS and MWS treatments, respectively. These results indicated that water stress may have affected berry development and led to a reduction in grape yield.

At the E-L 38 stage in 2021, the TSS and total anthocyanins content of the berries in the CK treatment were significantly lower than those in the LWS and MWS treatments; however, no significant changes were found between the LWS and MWS treatments. In 2022, the TSS and total anthocyanins content in the LWS treatment were 6.17% and 23.93% higher than in the CK treatment, respectively, while in the MWS treatment, they were 9.93% and 31.05% higher than in the CK treatment, respectively. As shown in Table 1, water stress caused a decrease in the TA content in 2021 and 2022. In 2022, the TA of the CK berries was 1.31 and 1.53 times higher than that of berries in the LWS and MWS treatments, respectively. The results showed that water stress reduced the titratable acid of the ‘Marselan’ fruits but increased the TSS and total anthocyanins content.

### 3.3. Analysis of the Main Organic Acids in Grape Berries under Water Stress

**Figure 3:**
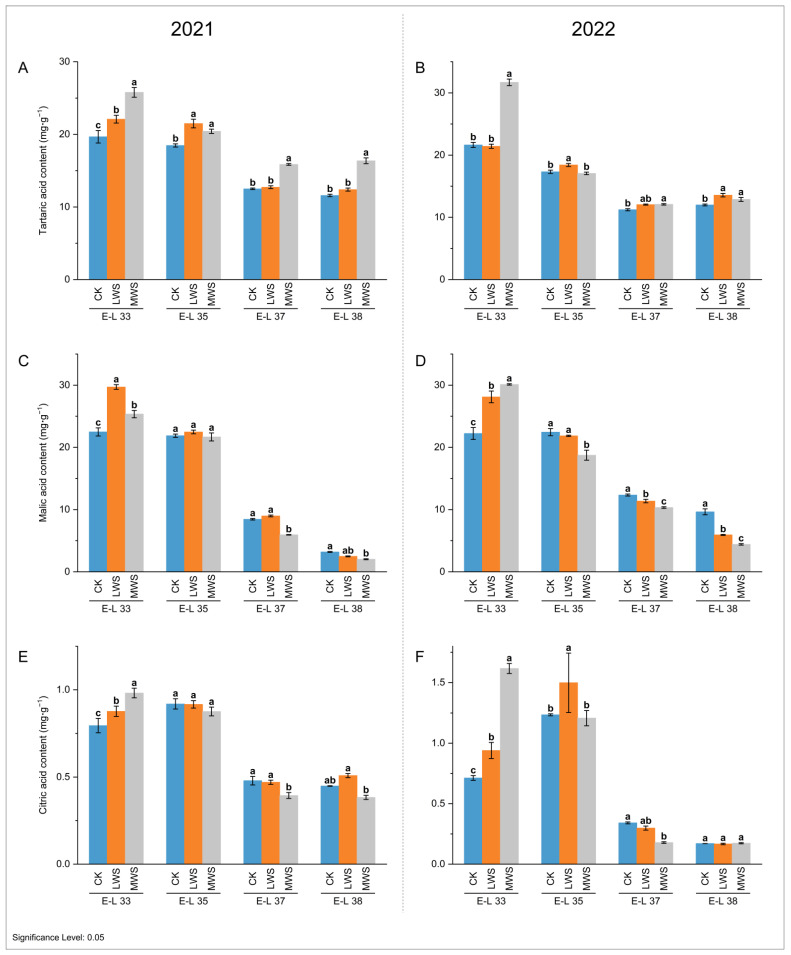
Effects of vine water status on the contents of tartaric acid (**A**,**B**), malic acid (**C**,**D**), and citric acid (**E**,**F**) in grape berries in 2021 and 2022. Different letters indicate statistically significant differences (*p* < 0.05, Tukey’s test).

The influence of the vine water status on the contents of three main organic acids (malic, tartaric, and citric acid) was evaluated in developing ‘Marselan’ berries from pre-veraison to harvest. As shown in Figure 3, tartaric and malic acid were the main organic acids. Over two consecutive years, these two organic acids showed a similar accumulation pattern, with an overall decreasing trend. After the E-L37 stage in 2021 and 2022, the tartaric acid content in the berries did not appear to decrease significantly over time (Figure 3A,B). Additionally, the tartaric acid content in the water stress treatments was consistently higher than that in the CK treatment.

At the E-L 33 stage, water stress significantly increased the malic acid content. In 2021, the malic acid content in the LWS treatment was 29.70 mg·g^−1^, which was 32.12% higher than that in the CK treatment. In 2022, the malic acid content in the MWS treatment reached 30.12 mg·g^−1^, which was 1.35 times that of the CK treatment (Figure 3C,D). No statistical difference was observed among the three treatments at the E-L 35 stage in 2021, whereas the malic acid content following the MWS treatment in 2022 was significantly lower than that following the CK treatment. At the E-L 38 stage in 2021 and 2022, the malic acid content following the MWS treatment was significantly reduced by 36.68% (2.02 mg·g^−1^) and 54.25% (4.41 mg·g^−1^), respectively, compared to that of the CK treatment. The variation trend in the citric acid content was similar to malic acid across all the berry development stages (Figure 3E,F). At the E-L 33 stage, the content of citric acid in the MSW treatment was 0.98 mg·g^−1^ in 2021, which was 22.50% higher than that of the CK treatment. In 2022, it was 1.62 mg·g^−1^, which was 128.17% higher than the control. However, the grape berries under the water stress in both years exhibited a similar concentration of citric acid (0.17–0.51 mg·g^−1^) at the E-L 38 stage.

### 3.4. Malic Acid Metabolic Enzyme Activity and Gene Expression under Water Stress

**Figure 4:**
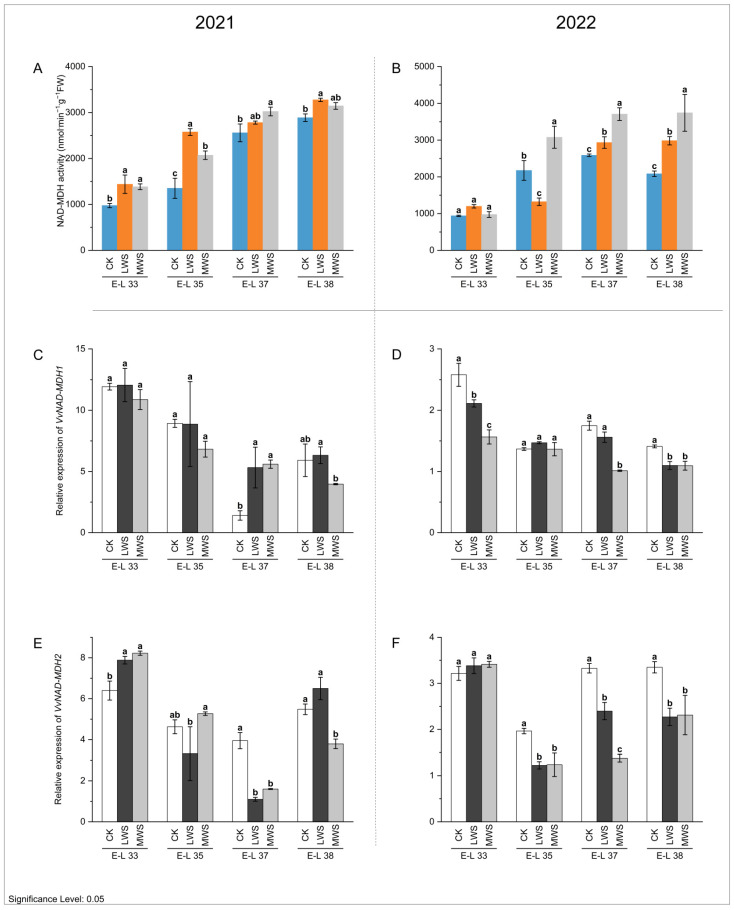
Effects of vine water status on the activities of NAD-MDH (**A**,**B**) and expression levels of *NAD-MDH* genes (**C**–**F**) in grapes during the berry ripening process. Different letters indicate statistically significant differences (*p* < 0.05, Tukey’s test). NAD-MDH, NAD-malate dehydrogenase.

Cytosolic malate dehydrogenase (MDH) is a crucial enzyme that facilitates the interconversion between malate and oxaloacetate (OAA) using NAD(P)H. The NAD-MDH enzyme activity gradually increased, reaching a peak at the E-L 38 stage in both 2021 and 2022 for all the treatments (Figure 4A,B). Water stress induced an overall increase in NAD-MDH activity compared to the control. During the E-L 38 stage, the NAD-MDH activity of the CK and LWS treatments in 2021 was 2887.8 and 3144.9 nmol·min^−1^·g^−1^ FW, respectively (Figure 4A). In 2022, the NAD-MDH activity of the LWS and MWS treatments was 43.2% and 79.6% higher than that of the CK treatment (Figure 4B). Water stress inhibited the expression of *VvNAD-MDH1/2* genes in the berries. *VvNAD-MDH1* gene expression in the CK treatment was 1.49 and 1.29 times higher than that in the MWS treatment during the E-L 38 stage in 2021 and 2022 (Figure 4C,D), respectively, and the *VvNAD-MDH2* gene expression was 2.41 and 1.45 times higher than that in the MWS treatment, respectively (Figure 4E,F).

**Figure 5:**
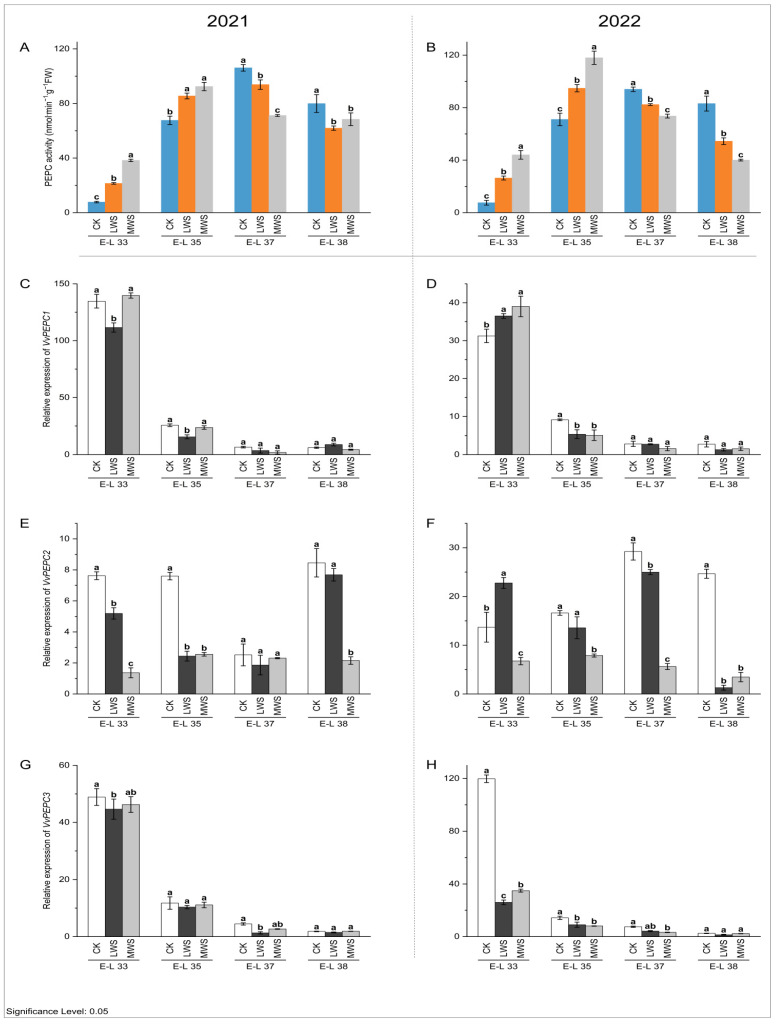
Effects of vine water status on the activities of PEPC (**A**,**B**) and expression levels of *PEPC* genes (**C**–**H**) in grapes during the berry ripening process. Different letters indicate statistically significant differences (*p* < 0.05, Tukey’s test). PEPC, Phosphoenolpyruvate carboxylase.

During grape berry growth and development, the highest activity of the PEPC enzyme was recorded at the E-L 35 stage (Figure 5A). Water stress had different effects on the PEPC enzyme activity of the grape berries at different berry developmental stages. At the E-L 33 and 35 stages, the enzyme activity was significantly increased compared to the activity in the control treatment. At the E-L 33 stage in 2021, the PEPC enzyme activities in the berries of the CK, LWS, and MWS treatments were 7.75, 21.42, and 38.28 nmol·min^−1^·g^−1^ FW, respectively, and for 2022, the PEPC enzyme activities in the berries of the CK, LWS, and MWS treatments were 7.53, 26.38, and 44.03 nmol·min^−1^·g^−1^ FW, respectively, for the same berry developmental stages. For the berries sampled at the E-L 37 and 38 stages, the PEPC enzyme activities were inhibited for the vines subjected to water stress. In particular, the PEPC enzyme activities in the MWS treatment in 2021 and 2022 were 14.39% and 53.73% lower than those of the control treatment, respectively (Figure 5A,B). At the same stage in 2022, the LWS treatment exhibited a 44.29% lower growth rate than the control treatment, as shown in Figure 5C, and there was no significant difference compared to the MWS treatment (Figure 5D). The expression of the *VvPEPC2* gene was significantly inhibited by water stress (Figure 5E), indicating that the *VvPEPC2* gene could play a role in malic acid metabolism in response to vine water status. At the E-L 38 stage in 2021 and 2022, the expression of *VvPEPC2* in the CK treatment was 3.93 and 7.11 times higher, respectively, than that in the MWS treatment (Figure 5E,F). Meanwhile, water stress significantly inhibited the expression of *VvPEPC3* at the E-L 33 and 37 stages; however, no effect occurred in the E-L 38 stage (Figure 5G,H).

**Figure 6:**
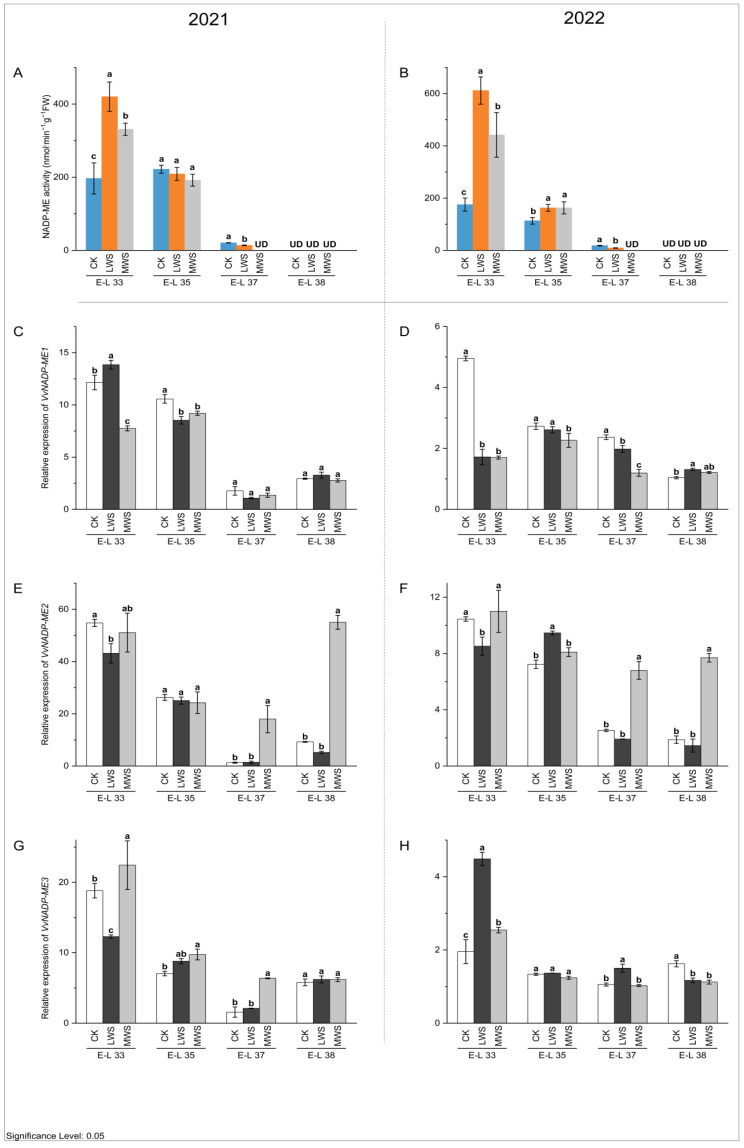
Effects of vine water status on the activities of NADP-ME (**A**,**B**) and expression levels of *NADP-ME* genes (**C**–**H**) in grapes during the berry ripening process. Different letters indicate statistically significant differences (*p* < 0.05, Tukey’s test). NADP-ME, NADP malic enzyme.

**Figure 7:**
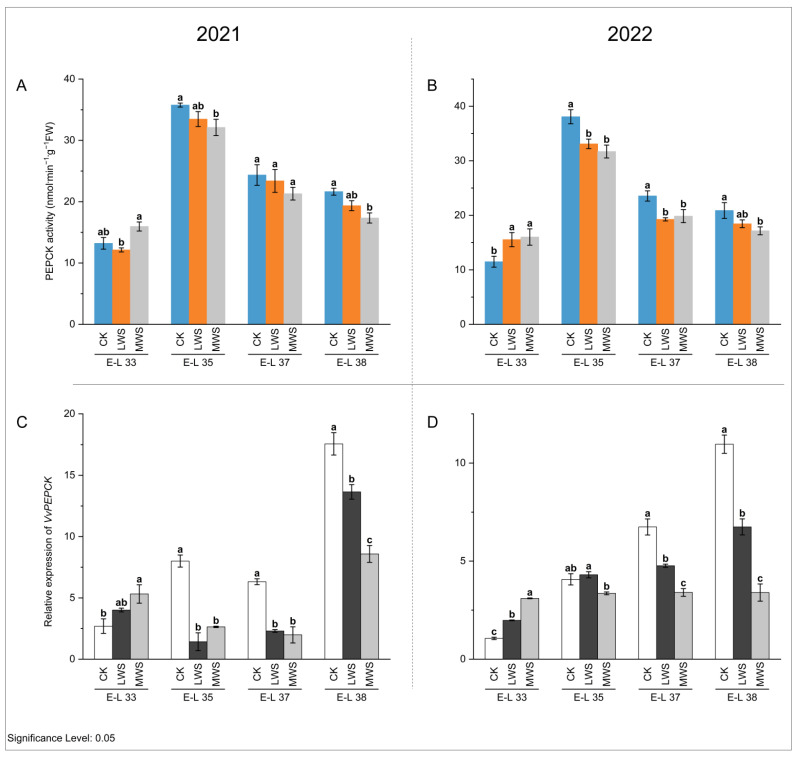
Effects of vine water status on the activities of PEPCK (**A**,**B**) and expression levels of *PEPCK* genes (**C**,**D**) in grapes during the berry ripening process. Different letters indicate statistically significant differences (*p* < 0.05, Tukey’s test). PEPCK, phosphoenolpyruvate carboxykinase.

NADP-ME and PEPCK are key enzymes involved in malic acid degradation in fruit [32]. The activity of the NADP-ME enzyme in berries in 2021 and 2022 showed a continuously decreasing trend, and the enzyme activity could not be detected at the E-L 38 stage (Figure 6A,B). At the E-L 33 stage, water stress induced an increase in the NADP-ME enzyme activity compared to the control. In 2021 and 2022, the NADP-ME enzyme activity was the highest in the LWS treatment, which was 2.14 and 3.50 times higher than that in the control treatment, respectively. However, at the E-L 37 stage, water stress inhibited NADP-ME enzyme activity, the LWS treatment was lower than the CK treatment, and NADP-ME enzyme activity could not be detected in the MWS treatment. These results indicated that the effects of water stress on NADP-ME enzyme activity in the ‘Marselan’ berries were different at the E-L 33 and 37 stages. Quantitative analysis (Figure 6C–H) showed that all three NADP-ME genes (*VvNADP-ME1*, *VvNADP-ME2*, and *VvNADP-ME3*) were expressed at the highest level at E-L 33, consistent with the activity dynamics of NADP-ME, indicating that the changing trend in the enzyme activity of NADP-ME was consistent with that of the gene expression patterns. The expression level of the *VvNADP-ME1* gene was the highest in the LWS treatment at the E-L 33 stage in 2021, but at the same stage in 2022 (Figure 6C), the CK treatment was significantly higher than in the water stress treatments (Figure 6D). The effects of water stress on *VvNADP-ME1* were not significant during E-L 37–38 in 2021, while it was significantly inhibited by water stress at the E-L 37 stage in 2022. Compared with the control treatment, the expression of *VvNADP-ME1* in LWS and MWS increased at the E-L 38 stage in 2022, but there was no significant difference between the water stress treatments. The expression of *VvNADP-ME2* was 2.68–14.03-fold higher in each MWS treatment compared to the CK treatment during the E-L 37–38 stage in 2021 and 2022 (Figure 6E,F). At the E-L 33 stage in 2021, the highest expression of *VvNADP-ME3* in the MWS treatment was 1.19 times higher than that in the CK treatment, while in 2022, the highest expression of this gene in the LWS treatment was 2.30 times higher than that in the CK treatment at the same stage (Figure 6G,H). In Figure 7A,B, it is shown that the PEPCK enzyme activity increased and then decreased during berry growth and development. Water stress induced an inhibitory effect on the PEPCK activity during the rest of the stage, except in the E-L 33 stage, where it increased. At the E-L 35 stage in both 2021 and 2022, the PEPCK enzyme activity was 11.3% and 20.1% higher in the CK treatment than in the MWS treatment, respectively, with no significant differences between the treatments. Furthermore, the *VvPEPCK* expression levels gradually increased during berry development for the studied developmental stages and reached its highest values in the ripe berries. The gene expression results were consistent with the previous results on enzyme activity. The expression level of the *VvPEPCK* gene in the MWS treatment was significantly higher than the control treatment in the E-L 33 stage of 2021 and was significantly inhibited by water stress in the other stages. At the E-L 38 stage, the expression level of the *VvPEPCK* gene following the CK treatment in 2021 was 1.29 and 2.04 times higher than that following the LWS and MWS treatments, respectively. At the same stage of 2022, the CK was 3.23 and 1.63 times that of the LWS and MWS treatments, respectively (Figure 7C,D).

**Figure 8:**
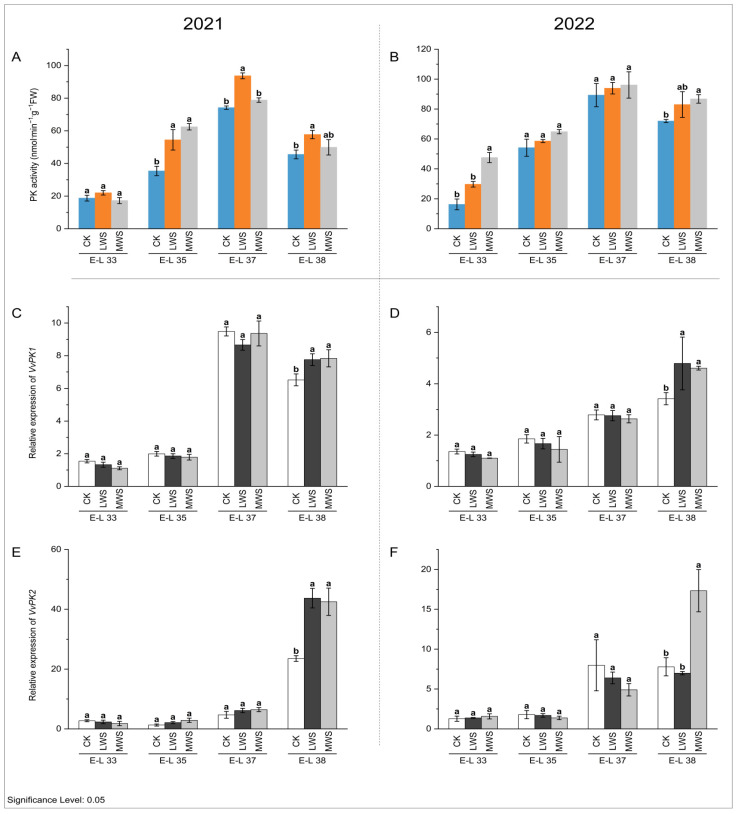
Effects of vine water status on the activities of PK (**A**,**B**) and expression levels of *PK* genes (**C**–**F**) in grapes during the berry ripening process. Different letters indicate statistically significant differences (*p* < 0.05, Tukey’s test). PK, Pyruvate dikinase.

Pyruvate dikinase (PK) is an essential enzyme involved in pyruvate metabolism, as it catalyzes the regeneration of phosphoenolpyruvate [26]. As depicted in Figure 8A,B, the PK activity exhibited an initial increase and subsequent decrease during berry development, with water stress generally stimulating its activity. Throughout fruit development, the expression levels of *VvPK1* and *VvPK2* in the grape berries gradually increased. Notably, water stress significantly augmented the transcription levels of *VvPK1* and *VvPK2* in the berries at the E-L 38 stage in both 2021 and 2022 (Figure 8C–F). Specifically, at the E-L 38 stage in 2021, the expression of *VvPK2* in the MWS treatment group was 1.81-fold higher than that in the control treatment group (Figure 8E), whereas there was no significant difference between the water stress treatments. In contrast, at the same stage in 2022, the expression of *VvPK2* in the MWS treatment was 2.23-fold greater than that in the control treatment (Figure 8F).

### 3.5. Differential Metabolites Affected by Water Stress

**Figure 9:**
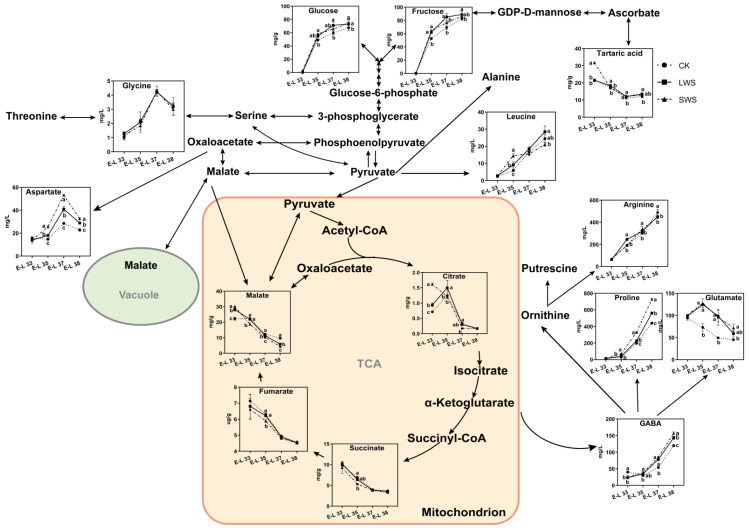
Schematic representations of altered metabolites in grape berries under different vine water status involved in the tricarboxylic acid (TCA) cycle. Different letters indicate statistically significant differences (*p* < 0.05, Tukey’s test).

**Figure 10:**
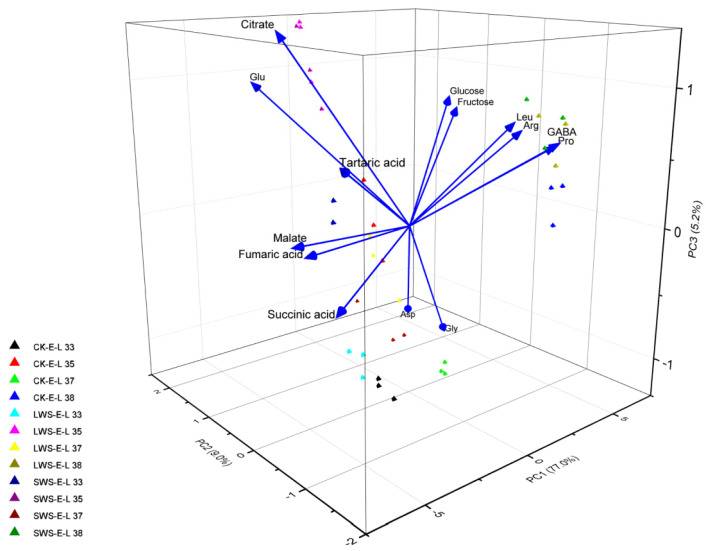
Principal components analysis (PCA) was performed using 14 compounds in the grape berries from the three treatment groups in 2022.

Figure 9 shows the change in the concentration of 14 metabolites in the CK and water stress treatments during berry development. The fructose and glucose levels were significantly enhanced in the berries exposed to water stress, especially at the E-L 38 stage. During the early stages of development, the concentration of amino acids in the grape berries was relatively low. However, as the berries matured, a significant increase in the concentration of these amino acids was observed. Arginine (Arg) and proline (Pro) are generally the most abundant amino acids in grapes, together with leucine (Leu), GABA, and glutamate (Glu). The GABA and Pro concentrations were significantly induced by the water stress. Moreover, the water stress also increased the concentration of Leu and aspartate (Asp), whereas no difference in the glycine (Gly) content was observed among the three treatments. Reductions in TCA cycle organic acids from the water stress treatments were observed, and lower fumarate and succinate levels were only found in the grape berries at the E-L 35 stage. To classify the different treatments, a principal component analysis (PCA) was performed using the concentration of metabolites obtained from the CK and water stress grapevines in 2022. The results are shown in Figure 10. Principal component 1 (PC1) explained 77.0% of the variance, and PC2 explained 9.0% of the variance, representing 86.0% of all the variance. PC1 separated the variations based on developmental stages, while PC2 separated them based on irrigation regimes. The separation of the three treatments in the PCA plots at the E-L 35 and 37 stages was greater than that at the E-L 33 and 38 stages, indicating that the water stress had a greater effect on the metabolites of the grape berries from the E-L 35 and 37 stages.

## 4. Discussion

The distribution of water resources shows a strong regional disparity in China. The scarcity of per capita water resources is particularly evident in the arid and semi-arid regions of the northwest [6]. Moreover, escalating global climate change could result in unpredictable patterns of precipitation and rising temperatures, potentially leading to more frequent droughts and amplifying the demand for freshwater resources. Practicing precision irrigation in vineyards is crucial for saving water while considering practical goals such as yield and the aromatic profiles of wine. It has been shown that a specific threshold of water stress can improve the quality of certain red wines [33,34,35]. While water restriction may reduce the fresh weight and total acid content of wine grapes, it has a positive effect on quality (increased concentration of primary and secondary metabolites) [6,8,36,37]. In this experiment, the berry composition at maturity was modified directly or indirectly by the vine water status; however, other abiotic factors could have played a role in the fruit zone microclimate in terms of light and temperature [38,39]. Restricting the water status of the vine did increase the berry sugar and anthocyanin content, while the berry weight and titratable acidity decreased. These findings are consistent with previous studies [4,6,7,8] and demonstrate that vine water status is important for vine and berry development and physiology. In that regard, dry land irrigation is needed for vine and vineyard sustainability and wine quality. This addresses the question of water availability and management between people and agriculture (more information is provided in the FAO reports).

Organic acid, particularly malic acid, is an important characteristic of fruit ripening and quality. Malic acid is the second main organic acid accumulated during the berry development of wine grapes, which accumulates during the berry green growth stage and decreases from the onset of veraison (berry softening) to the plateau of berry sugar accumulation [40]. Genotypes, environmental factors, and cultural practices affect the synthesis and metabolism of malic acid in grape berries, leading to significant changes in the organic acid content of berries from berry set to harvest. In a study, it was observed that the titratable acid content decreased at high temperatures [41]. This reduction could be attributed to the increased breakdown of malic acid, possibly because of its role in the respiration process of grape berries [42,43,44]. Different studies on various grape varieties have shown a similar decline in the malic acid content in grape berries as water availability decreases, especially after veraison [15,45]. The effect of water deficits on malic acid metabolism in grape berries is related to their developmental stage. Malic acid increased at 40 days after anthesis as a result of water deficits but decreased at 106 days after anthesis [46]. In this study, the malic acid content was increased before veraison by water stress; however, the malic acid in the berries of the water stress treatment group was significantly reduced after veraison (E-L 37, 38) (Figure 3C,D). This decrease in malic acid content is believed to be caused by the accelerated degradation of malic acid under water stress conditions.

A decrease in malate content is mainly involved in three metabolic pathways, namely, glycolysis, the TCA cycle, and gluconeogenesis [2]. Malic acid metabolism-related enzymes, including NAD-MDH (EC 1.1.1.37), PEPC (EC 4.1.1.31), NADP-ME (EC 1.1.1.40), PEPCK (EC 4.1.1.49), and PK (EC 4.2.1.3), potentially play an important role in the above three pathways [47]. MDHs are crucial in the reversible interconversion between malate and oxaloacetate (OAA) and are present in various forms in the cytosol, mitochondria, peroxisomes, and other organelles of cells, including chloroplasts that contain NADP-dependent MDH [48]. In apples, NAD-MDH activity gradually increases during growth and development, while other enzymes may also be involved in the metabolism of malic acid in mature fruit [49]. In strawberries, NAD-MDH activity increases during fruit development and is consistently involved in malic acid synthesis, which is related to the increasing respiration rate during ripening [50]. Malic acid is synthesized and catabolized in grape berries, while in green fruit, malic acid is mainly produced by unloading photoassimilates, and it accumulates in the vacuoles of fruit cells. NAD-MDH activity in ‘Marselan’ berries gradually increases from E-L 33 to 38, while the malic acid content in the berries begins to decline after reaching the highest level (Figure 4A,B). The MDH reaction is involved in the TCA cycle and redox homeostasis between organelle compartments, which can be located in the cytosol, where it is a part of gluconeogenesis [51]. Although the NAD-MDH enzyme is more inclined to the production of malic acid in vitro, whether OAA or malate form depends on the physiological conditions in vivo. The MDH activity in the grape berries did not positively correlate with the malate content during fruit ripening, suggesting it may play a role in gluconeogenesis during berry ripening or the roles of other enzymes in malate accumulation. These results also showed that the overall pattern of NAD-MDH activity in the water-stressed berries was similar to that in the control, and that water stress was significantly effective in enhancing enzyme activity. In Arabidopsis, cytosolic malate dehydrogenase is considered to regulate cell metabolism to adapt to environmental constraints [52]. Additionally, the *VvNAD-MDH* mRNAs oscillated in quite a different way compared to the NAD-MDH activity (Figure 4C–F). A possible explanation for this phenomenon is the existence of post-translational regulation of NAD-MDH activity. PEPC is a well-studied enzyme in the plant kingdom, which is regulated by cytoplasmic pH and internal metabolites, with malic acid being a common inhibitor [18]. The activity of PEPC in ‘Marselan’ berries increases gradually and then decreases gradually until ripening, which is consistent with findings in plums, pineapple, and loquat [53,54,55]. Studies have shown that the enzymatic activity of PEPC in grape berries changes significantly during growth and development and is influenced by environmental factors. For example, Diakou et al. [56] found that PEPC enzyme activity showed different trends during the growth and development of different grape varieties, such as ‘Cabernet Sauvignon’ and ‘Gora Chirine’. The PEPC activity in berries of *Vitis amurensis* was examined by Liu [57], who reported that the activity exhibited a low level during the initial stage of green fruit, progressively rose, attained its maximum at mid-veraison, and then sharply declined until the maturation of the berries. PEPC is involved in malic acid accumulation during grape berry development [26]. However, the lack of correlation between PEPC activity and malic acid concentration does not necessarily indicate that PEPC is not involved in the accumulation of malic acid during ‘Marselan’ fruit development. The accumulated malic acid could reduce PEPC activity through feedback inhibition. The activity of PEPC in berries decreased with heat treatment before veraison, but the change in malic acid content was not related to the activity of PEPC, indicating that PEPC was not the rate-limiting enzyme for malic acid accumulation before veraison [58]. Water stress significantly promoted PEPC activity in berries at the E-L 33 and 35 stages but significantly inhibited PEPC activity at the E-L 37 and 38 stages. The consistent effect of water stress on malic acid and PEPC activity in berries after veraison indicates that the PEPC enzyme is involved in the metabolism of malic acid in berries. This was also confirmed by studies on the effect of high-temperature treatment on malic acid and PEPC activity in grapevines after veraison [26]. At the transcriptional level, *VvPEPCs* tend to be expressed in the early stages of grape berry development [43]. In the study, the expression levels of *VvPEPC1/3* in ‘Marselan’ berries gradually decreased during berry ripening, and the expression level of *VvPEPC* genes was reduced by water stress. The genes encoding PEPC are considered to have obvious post-transcriptional modification mechanisms for controlling gene expression [32,59].

In apples, NADP-ME activity has been shown to have a negative correlation with organic acid content and is believed to play a significant role in the degradation of malic acid in the cytoplasm [60]. However, in grapes, NADP-ME is known to catalyze the carboxylation of pyruvate, fixing carbon dioxide to produce malic acid. This study found that NADP-ME activity and gene expression levels were highest at the E-L 33 stage and then decreased at a later stage, which is consistent with previous results. Similar trends were observed in the growth and development of loquats and plums [54]. Water stress significantly affected NADP-ME activity and gene expression levels at the E-L 33 stage, indicating that the NADP-ME enzyme is regulated by water stress during the malic acid accumulation stage. Thus, malic acid breakdown in grape berries is probably not controlled by the NADP-ME enzyme. The *VvNADP-ME* transcripts were consistent with the change in enzyme activity, and *VvNADP-ME2* in LWS grape berries was induced by water stress at the E-L 38 stage. The conversion of malic acid to pyruvic acid in grape berries is believed to be mainly caused by cytoplasmic NADP-ME, followed by pyruvate entering the TCA cycle. However, pyruvate may also be produced by PEPCK and PK. PEPCK and PK enzyme activity and gene expression were present throughout the development, but their activity and expression levels were higher at berry ripening. Water stress generally inhibited PEPCK enzyme activity and *VvPEPCK* gene expression levels. The effect of water stress on PK enzyme activity was opposite to that of PEPCK enzyme activity, promoting an increase in its enzymatic activity; however, its effect on *VvPK* gene expression levels was not significant. In plants, there are two pathways known to convert malate to sugars. One pathway utilizes PEPCK, and the other pathway utilizes pyruvate orthophosphate dikinase. This study provides evidence for the gluconeogenesis from malic acid in grape berries via the PEPCK pathway.

The TCA cycle provides both organic acids and precursors for the biosynthesis of amino acids [61]. In agreement with previous reports, proline and arginine were the most abundant amino acids in the grape berries [62]. Proline plays an important role in enhancing plant tolerance to environmental stress. An increased level of proline was detected after the water stress treatments in 2022, and the increase was significant. GABA, which is synthesized from glutamate and ultimately produces succinate in the GABA pathway, is an important bypass of the TCA cycle in plants [63]. It is well documented that GABA can be induced quickly and accumulated at higher levels due to stress in plants, such as drought and chilling stress, and that this accumulation is generally accompanied by higher alanine levels and lower glutamate levels [64,65]. This also occurred in the present study, in which significant increases in the GABA contents were observed under water stress during the berry development stages. We speculated that the water stress treatment enhanced the activity of the GABA pathway in the ‘Marselan’ berries during development. However, more evidence still needs to be provided through further research.

## 5. Conclusions

This study found that water stress promoted an increase in soluble solids and total anthocyanins content, while the malic acid content decreased during the development of ‘Marselan’ berries. The level of malic acid was regulated by the combined activities of NADP-ME, NAD-MDH, PEPC, PEPCK, and PK. Water stress may promote the degradation of malic acid through the PEPCK pathway, which could result in enhanced gluconeogenesis. However, the potential gluconeogenesis due to malic acid conversion represents a very small percentage of the berry sugar content, which is mainly provided by leaf photosynthesis. Additionally, water stress induced significant alterations in the levels of free amino acids, including GABA, proline, leucine, aspartate, and glutamate. Notably, glutamate and GABA are key metabolites in the GABA pathway. These results provide novel evidence for understanding the relationship between the vine water status and malic acid metabolism in grape berries. Understanding the mechanisms affecting malic acid biosynthesis and degradation in grape berries is crucial for various purposes, including grapevine genetic selection. This knowledge could improve vineyard cultural practices, aid in selecting varieties with a higher level of organic acids for semi-arid and warm–hot wine regions, and facilitate adjustments to the oenological process.

## Figures and Tables

**Table 1:** Effects of vine water status on the fruit composition of *Vitis vinifera* L. cv. Marselan at the E-L 38 stage. Values are expressed as the mean ± standard deviation (SD). Different letters indicate statistically significant differences. (*p* < 0.05, Tukey’s test).

Treatment		Berry Fresh Mass (g) of 50 Berries	TSS (°Brix)	Titratable Acidity (g/L)	Total Anthocyanins Content (mg/g)
2021	CK	50.79 ± 1.39 a	22.77 ± 0.12 b	9.41 ± 0.37 a	3.49 ± 0.24 b
	LWS	36.11 ± 0.86 b	23.27 ± 0.09 a	7.41 ± 0.24 b	4.29 ± 0.28 a
	MWS	30.39 ± 0.49 c	23.81 ± 0.93 a	7.22 ± 0.22 b	4.44 ± 0.31 a
2022	CK	60.20 ± 0.39 a	24.77 ± 0.45 c	8.40 ± 0.21 a	4.22 ± 0.30 c
	LWS	50.79 ± 1.18 b	26.30 ± 0.33 b	6.39 ± 0.17 b	5.23 ± 0.07 b
	MWS	46.53 ± 0.80 c	27.23 ± 0.25 a	5.48 ± 0.32 c	6.12 ± 0.33 a

## Data Availability

The data that support this study are available in the article and the accompanying online supplementary materials.

## Supplementary Materials

The following supporting information can be downloaded at: https://www.mdpi.com/article/10.3390/foods12234191/s1, Figure S1: High-performance liquid chromatography (HPLC) chromatograms of the derivatives of amino acids, ammonium ion and biogenic amines at 280 nm, a standard solution (A) and γ-aminobutyric Acid (GABA) (B); Table S1: Irrigation amount of the three treatments; Table S2: List of primers for Real-Time PCR.

## References

[1] Umer M.J., Bin Safdar L., Gebremeskel H., Zhao S., Yuan P., Zhu H., Kaseb M.O., Anees M., Lu X., He N. (2020). Identification of key gene networks controlling organic acid and sugar metabolism during watermelon fruit development by integrating metabolic phenotypes and gene expression profiles. Hortic. Res..

[2] Sweetman C., Deluc L.G., Cramer G.R., Ford C.M., Soole K.L. (2009). Regulation of malate metabolism in grape berry and other developing fruits. Phytochemistry.

[3] Zhang X., Kontoudakis N., Šuklje K., Antalick G., Blackman J.W., Rutledge D.N., Schmidtke L.M., Clark A.C. (2020). Changes in Red Wine Composition during Bottle Aging: Impacts of Grape Variety, Vineyard Location, Maturity, and Oxygen Availability during Aging. J. Agric. Food Chem..

[4] Levin A.D., Deloire A., Gambetta G.A. (2020). Does water deficit negatively impact wine grape yield over the long-term?. IVES Tech. Rev..

[5] Wei Z., Luo J., Huang Y., Guo W., Zhang Y., Guan H., Xu C., Lu J. (2017). Profile of Polyphenol Compounds of Five Muscadine Grapes Cultivated in the United States and in Newly Adapted Locations in China. Int. J. Mol. Sci..

[6] Yang B., Yao H., Zhang J.X., Li Y., Fang Y. (2020). Effect of regulated deficit irrigation on the content of soluble sugars, organic acids and endogenous hormones in cabernet sauvignon in the ningxia region of china. Food Chem..

[7] Leng F., Zhou J., Wang C., Sun L., Zhang Y., Li Y., Wang L., Wang S., Zhang X., Xie Z. (2022). Post-veraison different frequencies of water deficit strategies enhance Reliance grapes quality under root restriction. Food Chem..

[8] Ma W., Lu S., Li W., Nai G., Ma Z., Li Y., Chen B., Mao J. (2023). Transcriptome and metabolites analysis of water-stressed grape berries at different growth stages. Physiol. Plant..

[9] Jan N., Anjum S., Wani S.M., Mir S.A., Malik A.R., Wani S.A., Hussein D.S., Rasheed R.A., Gatasheh M.K. (2022). Influence of Canning and Storage on Physicochemical Properties, Antioxidant Properties, and Bioactive Compounds of Apricot (*Prunus armeniaca* L.) Wholes, Halves, and Pulp. Front. Nutr..

[10] Bigard A., Romieu C., Sire Y., Veyret M., Ojeda H., Torregrosa L. (2019). The kinetics of grape ripening revisited through berry density sorting. Oeno One.

[11] Wang H., Miao Y., Xu X., Ye P., Wu H., Wang B., Shi X. (2022). Effects of Blending on Phenolic, Colour, Antioxidant and Aroma Components of Cabernet Sauvignon Wine from Xinjiang (China). Foods.

[12] Zhang F., Zhong H., Zhou X., Pan M., Xu J., Liu M., Wang M., Liu G., Xu T., Wang Y. (2022). Grafting with rootstocks promotes phenolic compound accumulation in grape berry skin during development based on integrative multi-omics analysis. Hortic. Res..

[13] Ma B., Yuan Y., Gao M., Li C., Ogutu C., Li M., Ma F. (2018). Determination of Predominant Organic Acid Components in Malus Species: Correlation with Apple Domestication. Metabolites.

[14] Guo C., Wang P., Zhang J., Guo X., Mu X., Du J. (2022). Organic acid metabolism in Chinese dwarf cherry [*Cerasus humilis* (Bge.) Sok.] is controlled by a complex gene regulatory network. Front. Plant Sci..

[15] Uriarte D., Intrigliolo D.S., Mancha L.A., Valdés E., Gamero E., Prieto M.H. (2016). Combined effects of irrigation regimes and crop load on ‘Tempranillo’ grape composition. Agric. Water Manag..

[16] Gambetta G.A., Herrera J.C., Dayer S.F., Feng Q., Hochbergm U., Castellarin S.D. (2020). The physiology of drought stress in grapevine: Towards an integrative definition of drought tolerance. J. Exp. Bot..

[17] Chen Y., Zeng L., Liao Y., Li J., Zhou B., Yang Z., Tang J. (2020). Enzymatic Reaction-Related Protein Degradation and Proteinaceous Amino Acid Metabolism during the Black Tea (*Camellia sinensis*) Manufacturing Process. Foods.

[18] Araujo W.L., Nunes-Nesi A., Sweetlove L.J., Fernie A.R. (2012). Metabolic control and regulation of the tricarboxylic acid cycle in photosynthetic and heterotrophic plant tissues. Plant Cell Environ..

[19] Sheteiwy M.S., Shao H., Qi W., Hamoud Y.A., Shaghaleh H., Khan N.U., Yang R., Tang B. (2019). GABA-Alleviated Oxidative Injury Induced by Salinity, Osmotic Stress and their Combination by Regulating Cellular and Molecular Signals in Rice. Int. J. Mol. Sci..

[20] Wang Z.P., Deloire A., Carbonneau A., Federspiel B., Lopez F. (2003). An in vivo experimental system to study sugar phloem unloading in ripening grape berries during water deficiency stress. Ann. Bot..

[21] Coombe B.G. (1995). Adoption of a system for identifying grapevine growth stages. Aust. J. Grape Wine Res..

[22] Deloire A., Pellegrino A., Rogiers S. (2020). A few words on grapevine leaf water potential. IVES Tech. Rev. Vine Wine.

[23] Lian T.T., Moe M.M., Kim Y.J., Bang K.S. (2019). Effects of Different Colored LEDs on the Enhancement of Biologically Active Ingredients in Callus Cultures of *Gynura procumbens* (Lour.) Merr. Molecules.

[24] Xu L., Yue Q., Bian F., Sun H., Zhai H., Yao Y. (2017). Melatonin enhances phenolics accumulation partially via ethylene signaling and resulted in high antioxidant capacity in grape berries. Front. Plant Sci..

[25] Wu Z.F., Tu M.M., Yang X.P., Xu J.H., Yu Z.F. (2020). Effect of cutting and storage temperature on sucrose and organic acids metabolism in postharvest melon fruit. Postharvest Biol. Technol..

[26] Sweetman C., Sadras V.O., Hancock R.D., Soole K.L., Ford C.M. (2014). Metabolic effects of elevated temperature on organic acid degradation in ripening *Vitis vinifera* fruit. J. Exp. Bot..

[27] Hirai M., Ueno I. (1977). Development of citrus fruits: Fruit development and enzymatic changes in juice vesicle tissue. Plant Cell Physiol..

[28] Han S., Nan Y., Qu W., He Y., Ban Q., Lv Y., Rao J. (2018). Exogenous γ-Aminobutyric Acid Treatment That Contributes to Regulation of Malate Metabolism and Ethylene Synthesis in Apple Fruit during Storage. J. Agric. Food Chem..

[29] Zhan Z.N., Wang N., Chen Z.M., Zhang Y.X., Geng K.Q., Li D.M., Wang Z.P. (2023). Effects of water stress on endogenous hormones and free polyamines in different tissues of grapevines (*Vitis vinifera* L. cv. ‘Merlot’). Funct. Plant Biol..

[30] Wang Y.Q., Ye D.Q., Zhu B.Q., Wu G.F., Duan C.Q. (2014). Rapid HPLC analysis of amino acids and biogenic amines in wines during fermentation and evaluation of matrix effect. Food Chem..

[31] Livak K.J., Schmittgen T.D. (2001). Analysis of relative gene expression data using real-time quantitative PCR and the 2(-Delta Delta C(T)) Method. Methods.

[32] Etienne A., Génard M., Lobit P., Mbeguié-A-Mbéguié D., Bugaud C. (2013). What controls fleshy fruit acidity? A review of malate and citrate accumulation in fruit cells. J. Exp. Bot..

[33] Garrido I., Uriarte D., Hernández M., Llerena J.L., Valdés M.E., Espinosa F. (2016). The Evolution of Total Phenolic Compounds and Antioxidant Activities during Ripening of Grapes (*Vitis vinifera* L., cv. Tempranillo) Grown in Semiarid Region: Effects of Cluster Thinning and Water Deficit. Int. J. Mol. Sci..

[34] Picard M., van Leeuwen C., Guyon F., Gaillard L., de Revel G., Marchand S. (2017). Vine Water Deficit Impacts Aging Bouquet in Fine Red Bordeaux Wine. Front. Chem..

[35] Yu R., Kurtural S.K. (2020). Proximal Sensing of Soil Electrical Conductivity Provides a Link to Soil-Plant Water Relationships and Supports the Identification of Plant Water Status Zones in Vineyards. Front. Plant Sci..

[36] Pellegrino A., Clingeleffer P., Cooley N., Walker R. (2014). Management practices impact vine carbohydrate status to a greater extent than vine productivity. Front. Plant Sci..

[37] Blancquaert E.H., Oberholster A., Ricardo-Da-Silva J.M., Deloire A.J. (2018). Effects of Abiotic Factors on Phenolic Compounds in the Grape Berry—A Review. S. Afr. J. Enol. Vitic..

[38] Deloire A., Pellegrino A. (2021). Review of vine water deficit. What levers for the vineyard in the short and medium term?. IVES Tech. Rev. Vine Wine.

[39] Wang X., Tu M., Wang D., Liu J., Li Y., Li Z., Wang Y., Wang X. (2018). CRISPR/Cas9-mediated efficient targeted mutagenesis in grape in the first generation. Plant Biotechnol. J..

[40] Shahood R., Torregrosa L., Savoi S., Romieu C. (2020). First quantitative assessment of growth, sugar accumulation and malate breakdown in a single ripening berry. Oeno One.

[41] Ruffner H.P., Hawker J.S., Hale C.R. (1976). Temperature and enzymic control of malate metabolism in berries of vitis vinifera. Phytochemistry.

[42] Ford C.M., Geròs H., Chaves M.M., Delrot S. (2012). The Biochemistry of organic acids in the Grape. The Biochemistry of the Grape Berry.

[43] Rienth M., Torregrosa L., Sarah G., Ardisson M., Brillouet J.M., Romieu C. (2016). Temperature desynchronizes sugar and organic acid metabolism in ripening grapevine fruits and remodels their transcriptome. BMC Plant Biol..

[44] Lecourieux F., Kappel C., Pieri P., Charon J., Pillet J., Hilbert G., Renaud C., Gomès E., Delrot S., Lecourieux D. (2017). Dissecting the Biochemical and Transcriptomic Effects of a Locally Applied Heat Treatment on Developing Cabernet Sauvignon Grape Berries. Front. Plant Sci..

[45] Cooley N.M., Clingeleffer P.R., Walker R.R. (2017). Effect of water deficits and season on berry development and composition of Cabernet Sauvignon (*Vitis vinifera* L.) grown in a hotclimate. Aust. J. Grape Wine Res..

[46] Savoi S., Wong D.C.J., Degu A., Herrera J.C., Bucchetti B., Peterlunger E., Fait A., Mattivi F., Castellarin S.D. (2017). Multi-Omics and Integrated Network Analyses Reveal New Insights into the Systems Relationships between Metabolites, Structural Genes, and Transcriptional Regulators in Developing Grape Berries (*Vitis vinifera* L.) Exposed to Water Deficit. Front. Plant Sci..

[47] Ali M.M., Anwar R., Rehman R.N.U., Ejaz S., Ali S., Yousef A.F., Ercisli S., Hu X., Hou Y., Chen F. (2022). Sugar and acid profile of loquat (*Eriobotrya japonica* Lindl.), enzymes assay and expression profiling of their metabolism-related genes as influenced by exogenously applied boron. Front. Plant Sci..

[48] Chen Y., Fu Z., Zhang H., Tian R., Yang H., Sun C., Wang L., Zhang W., Guo Z., Zhang X. (2020). Cytosolic malate dehydrogenase 4 modulates cellular energetics and storage reserve accumulation in maize endosperm. Plant Biotechnol. J..

[49] Yao Y.X., Li M., Zhai H., You C.X., Hao Y.J. (2011). Isolation and characterization of an apple cytosolic malate dehydrogenase gene reveal its function in malate synthesis. J. Plant Physiol..

[50] Iannetta P.P., Escobar N.M., Ross H.A., Souleyre E.J., Hancock R.D., Witte C.P., Davies H.V. (2010). Identification, cloning and expression analysis of strawberry (*Fragaria* x *ananassa*) mitochondrial citrate synthase and mitochondrial malate dehydrogenase. Physiol. Plant..

[51] Wang Q.J., Sun H., Dong Q.L., Sun T.Y., Jin Z.X., Hao Y.J., Yao Y.X. (2016). The enhancement of tolerance to salt and cold stresses by modifying the redox state and salicylic acid content via the cytosolic malate dehydrogenase gene in transgenic apple plants. Plant Biotechnol. J..

[52] Huang J., Niazi A.K., Young D., Rosado L.A., Vertommen D., Bodra N., Abdelgawwad M.R., Vignols F., Wei B., Wahni K. (2018). Self-protection of cytosolic malate dehydrogenase against oxidative stress in Arabidopsis. J. Exp. Bot..

[53] Jiang C.C., Fang Z.Z., Zhou D.R., Pan S.L., Ye X.F. (2019). Changes in secondary metabolites, organic acids and soluble sugars during the development of plum fruit cv. ‘Furongli’ (*Prunus salicina* Lindl). J. Sci. Food Agric..

[54] Chen F.X., Liu X.H., Chen L.S. (2009). Developmental changes in pulp organic acid concentration and activities of acid-metabolising enzymes during the fruit development of two loquat (*Eriobotrya japonica* Lindl.) cultivars differing in fruit acidity. Food Chem..

[55] Zhang X.M., Du L.Q., Sun G.M., Gong D.Q., Chen J.Y., Li W.C., Xie J.H. (2007). Changes in organic acid concentrations and the relative enzyme activities during the development of Cayenne pineapple fruit. J. Fruit Sci..

[56] Diakou P., Svanella L., Raymond P., Gaudillère J.P., Moing A. (2000). Phosphoenolpyruvate carboxylase during grape berry development: Protein level, enzyme activity and regulation. Aust. J. Plant Physiol..

[57] Liu L.Y. (2016). Study on the Accumulation Rule and Metabolic Regulation Mechanism of Glucuronic Acid. Ph.D. Thesis.

[58] Ruffner H.P. (1982). Metabolism of tartaric and malic acids in Vitis: A review—Part B. Vitis.

[59] Or E., Baybik J., Sadka A., Saks Y. (2000). Isolation of mitochondrial malate dehydrogenase and phosphoenolpyruvate carboxylase cDNA clones from grape berries and analysis of their expression pattern throughout berry development. J. Plant Physiol..

[60] Yao Y.X., Li M., Liu Z., You C.X., Wang D.M., Zhai H., Hao Y.J. (2009). Molecular cloning of three malic acid related genes MdPEPC, MdVHA-A, MdcyME and their expression analysis in apple fruits. Sci. Hortic..

[61] Hang J., Chen Y., Liu L., Chen L., Fang J., Wang F., Wang M. (2022). Antitumor effect and metabonomics of niclosamide micelles. J. Cell. Mol. Med..

[62] Teixeira A., Martins V., Noronha H., Eiras-Dias J., Gerós H. (2014). The first insight into the metabolite profiling of grapes from three *Vitis vinifera* L. cultivars of two controlled appellation (DOC) regions. Int. J. Mol. Sci..

[63] Clément G., Moison M., Soulay F., Reisdorf-Cren M., Masclaux-Daubresse C. (2018). Metabolomics of laminae and midvein during leaf senescence and source-sink metabolite management in *Brassica napus* L. leaves. J. Exp. Bot..

[64] Yong B., Xie H., Li Z., Li Y.P., Zhang Y., Nie G., Zhang X.Q., Ma X., Huang L.K., Yan Y.H. (2017). Exogenous Application of GABA Improves PEG-Induced Drought Tolerance Positively Associated with GABA-Shunt, Polyamines, and Proline Metabolism in White Clover. Front. Physiol..

[65] Ngaffo Mekontso F., Duan W., Cisse E., Chen T., Xu X. (2021). Alleviation of Postharvest Chilling Injury of Carambola Fruit by γ-aminobutyric Acid: Physiological, Biochemical, and Structural Characterization. Front. Nutr..

