# Supplementary material for: Effects of Vine Water Status on Malate Metabolism and γ-Aminobutyric Acid (GABA) Pathway-Related Amino Acids in Marselan (Vitis vinifera L.) Grape Berries
Source: Foods. 2023 Nov 21;12(23):4191. doi: 10.3390/foods12234191 (PMC10706070; doi:10.3390/foods12234191)
Supplement: Supplementary file 1 [file foods-12-04191-s001.zip › foods-2674381-supplementary.pdf]

Supplementary Table S1 Irrigation amount of the three treatments

Year	Month	Rainfall amount	CK		LWS		MWS	
			Amount of irrigation（L/plant）	Total irrigation water（L/plant）	Amount of irrigation（L/plant）	Total irrigation water（L/plant）	Amount of irrigation（L/plant）	Total irrigation water（L/plant）
2021	6*	16.854	16.8	182.9	9.4	107	4.8	50.4
	7	6.082	85.4		51.3		22.6	
	8	26.478	51.9		33.5		17.8	
	9	59.631	28.8		12.8		5.2	
2022	6*	30.410	5.5	106.8	1.8	48.6	0	25.3
	7	57.250	30.4		10.1		3.5	
	8	53.308	26.0		13.9		6.5	
	9	13.882	44.9		22.8		15.3	

*The amount of irrigation water was determined using a stress test conducted on June 18, 2021, and subsequently on June 26, 2022.

Supplementary Table S2 List of primers for Real-Time PCR

Gene name	Primer	Sequence (5’-3’)
VvGADPH	F	CACGGTCAGTGGAAGCATCAT
	R	CCTTGTCAGTGAACACACCAG
VvNAD-MDH1	F	ATTGGTGGATGCTGCTTTTC
	R	GCCTGGGACTTGTAGATGGA
VvNAD-MDH2	F	GTGATCACATACGCGATTGG
	R	CTGTGGCATCCATCTTTCCT
VvNADP-ME1	F	TGCTGTCAAGGTGATCAAGC
	R	AAAAATTGCACGACCCTGAG
VvNADP-ME2	F	TACCAAGGAGGTGGTTGAGG
	R	GGCACAAAAAGTTTGCCATT
VvNADP-ME3	F	CGGTCTGGGTTTGATCATCT
	R	CATACGCTTTAGCAGCCACA
VvPEPC1	F	TGCTAGGCATGCTGAAACAC
	R	GCAACTCGGAAGGTATCCAA
VvPEPC2	F	GTGCCTGCAAAAGTGTCTGA
	R	CAACTTCTTGGGGTCGTGTT
VvPEPC3	F	TCGGATCCTATCGAGAATGG
	R	GCGGTTGCCATTGAGATTAT
VvPEPCK	F	GTGACAATGGCGTGTCAAAC
	R	GGGATAGGATGCACGAGTGT
VvPK1	F	ATGGCGAATATAGACATCGAG
	R	CCTCGATGTCTATATTCGCCAT
VvPK2	F	ATGGCGATGGAGAAGAAAACG
	R	CGTTTTCTTCTCCATCGCCAT

A

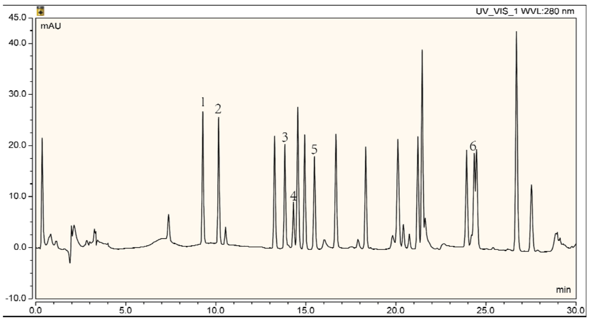

B

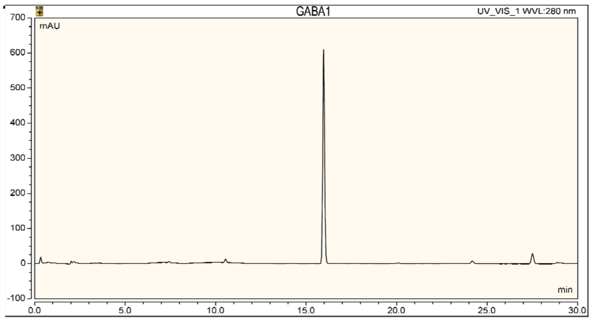

Supplementary Figure S1 High-performance liquid chromatography (HPLC) chromatograms of the derivatives of amino acids, ammonium ion and biogenic amines at 280 nm, a standard solution (A) and γ-aminobutyric Acid (GABA) (B). Peak assignments of the **Figure S1A**: (1) Asp; (2) Glu; (3) Gly; (4) Arg; (5) Pro.
